# Validation of CLASS MRI for personalized ACL footprints identification

**DOI:** 10.1002/ksa.12555

**Published:** 2024-12-15

**Authors:** Grégoire Thürig, Marc Barrera Usó, Raúl Panadero‐Morales, Julien Galley, Joseph Schwab, Alexander Heimann, Moritz Tannast, Daniel Petek

**Affiliations:** ^1^ Department of Orthopaedic Surgery and Traumatology Hospital and University of Fribourg Fribourg Switzerland; ^2^ Department of Orthopaedic Surgery and Traumatology Cantonal Hospital Schaffhausen Schaffhausen Switzerland; ^3^ Biomechanics Institute of Valencia Universitat Politècnica de València Valencia Spain; ^4^ Department of Radiology Hospital and University of Fribourg Fribourg Switzerland; ^5^ Department of Orthopaedic Surgery and Traumatology University Hospital of Bern Bern Switzerland

**Keywords:** ACL, anatomic reconstruction, anterior cruciate ligament, CLASS, graft positioning, MRI

## Abstract

**Purpose:**

In modern anterior cruciate ligament (ACL) surgery, the focus is usually on anatomical reconstruction to restore the natural kinematics of the knee. The individual optimal positioning of the ACL footprints (FPs) in primary surgery is still controversial and, especially in revision surgery, difficult to realize surgically. In this regard, a new MRI‐based sequence, the Compressed Lateral and anteroposterior Anatomic Systematic Sequence (CLASS) with marked femoral and tibial FPs as a template, could help. The purpose of this study was to (1) validate the reliability and reproducibility of the localization of femoral and tibial FPs of ACL in the generation of CLASS and (2) compare the identification of ACL FPs by CLASS with previously described methods.

**Methods:**

Magnetic resonance imaging (MRI) of uninjured knees from a predominantly young cohort is used to apply the CLASS algorithm. ACL FPs were subsequently identified by a board‐certified radiologist and an orthopaedic knee surgeon. Intraobserver reliability and interobserver reproducibility were assessed. Measurements of the ACL FPs according to established methods were performed and compared with the results from the literature.

**Results:**

Identification of ACL FPs and generation of CLASS images resulted in ‘almost perfect’ reliability and reproducibility. Most measurements also showed ‘almost perfect’ consistency. Statistical analysis showed significant variations between the deep‐shallow and high‐low positions when compared to the published literature.

**Conclusions:**

The CLASS MRI sequence is a reliable and reproducible method for identifying ACL FPs. The observed variability in the location of the ACL FP underlines the importance of a patient‐specific surgical approach.

**Level of Evidence:**

Level II.

**Abbreviations**
ACL: anterior cruciate ligament
AP: anteroposterior
CI: confidence interval
CLASS: Compressed Lateral and anteroposterior Anatomical Systematic Sequence
DS: deep‐shallow
FP: footprint
HL: high‐low
ICC: intraclass correlation coefficient
LAT: lateral
MRI: magnetic resonance imaging

## INTRODUCTION

The aim of today's anterior cruciate ligament (ACL) reconstructions is to achieve anatomical graft placement in order to mimic a normal knee kinematic [2, 22]. It is often difficult to accurately replicate the native ACL footprint (FP), especially when reconstructing with only one bundle. As a result, there was a consensus that reconstruction of the anteromedial (AM) bundle is paramount, especially with regard to the tunnel position on the femoral side [1, 13]. Biomechanical evidence further supports this and suggests that centring the graft on the AM bundle FP rather than on a centralized FP or the posterolateral (PL) bundle FP leads to better graft isometry [16, 27, 34, 50]. However, the clinical results of a study comparing centralized with AM graft placement showed no significant differences [51]. However, the debate on optimal graft positioning remains open [8, 25, 41].

Recent studies have highlighted the inherent variability in individual ACL FPs, which complicates the standardization of surgical techniques. The literature presents various methodologies to accurately assess femoral and tibial ACL FP [18, 36]. Recent studies have validated the use of three‐dimensional magnetic resonance imaging (3D‐MRI) to identify the ACL FP [5, 15, 43]. This has led to the development of new imaging modalities aimed at improving the accuracy and reliability of FP identification [46]. The Compressed Lateral and anteroposterior Anatomical Systematic Sequence (CLASS) compresses 3D‐MRI data into lateral and anteroposterior views and facilitates the projection of identified femoral and tibial ACL FP centroids onto accurate anteroposterior and sagittal knee views [46]. By using the CLASS MRI sequence, this research attempts to bridge the gap between advanced imaging techniques and the anatomical details of the ACL. The generation of a template in a clinical setting could enhance the reproducibility of patient‐specific anatomical identification and improve intraoperative tunnel positioning accuracy, particularly benefiting less experienced surgeons. Moreover, in ACL revision surgeries where anatomical references may be altered, such templates could significantly contribute to safer and more precise tunnel placement, potentially improving surgical outcomes and reducing complications.

The aim of this study was to (1) validate the reliability and reproducibility of the localization of femoral and tibial FPs in the generation of CLASS and (2) compare the identification of ACL FPs by CLASS with previously described methods. The hypothesis was that CLASS demonstrates the variability in individual ACL FPs, providing a reliable and reproducible template that could enhance patient‐specific surgical approaches.

## MATERIALS AND METHODS

All MRI procedures of the knee have been reviewed that had contemporary (i.e., within 1 month) anteroposterior and lateral plain radiographs performed at the Fribourg Cantonal Hospital between 2015 and 2021. These imaging datasets were subsequently stored and accessed through the institution's Picture Archiving and Communication System (PACS, GE Healthcare). A total number of 2462 MRIs of patients from 18 to 65 years old were available for inclusion. MRIs showing substantial pathological changes to the knee (*n* = 645), a history of ACL lesion (*n* = 472), or other traumatic injuries (*n* = 1263) were excluded. After these exclusion criteria were applied, a total number of 82 patients (82 knees) were available for further analysis. The study population comprised a predominantly Caucasian cohort, consisting of 44 females and 38 males. It's noteworthy to mention that these 82 MRI scans were primarily commissioned by general practitioners in response to a diverse range of patient complaints. None of these cases required further evaluation by an orthopaedic specialist.

### CLASS MRI

All knee MRIs were acquired using a standardized MR‐technique and an Optima MR360 1.5 T Advance scan, GE Healthcare, or a Discovery MR750 3 T, GE Healthcare.

**Figure 1:**
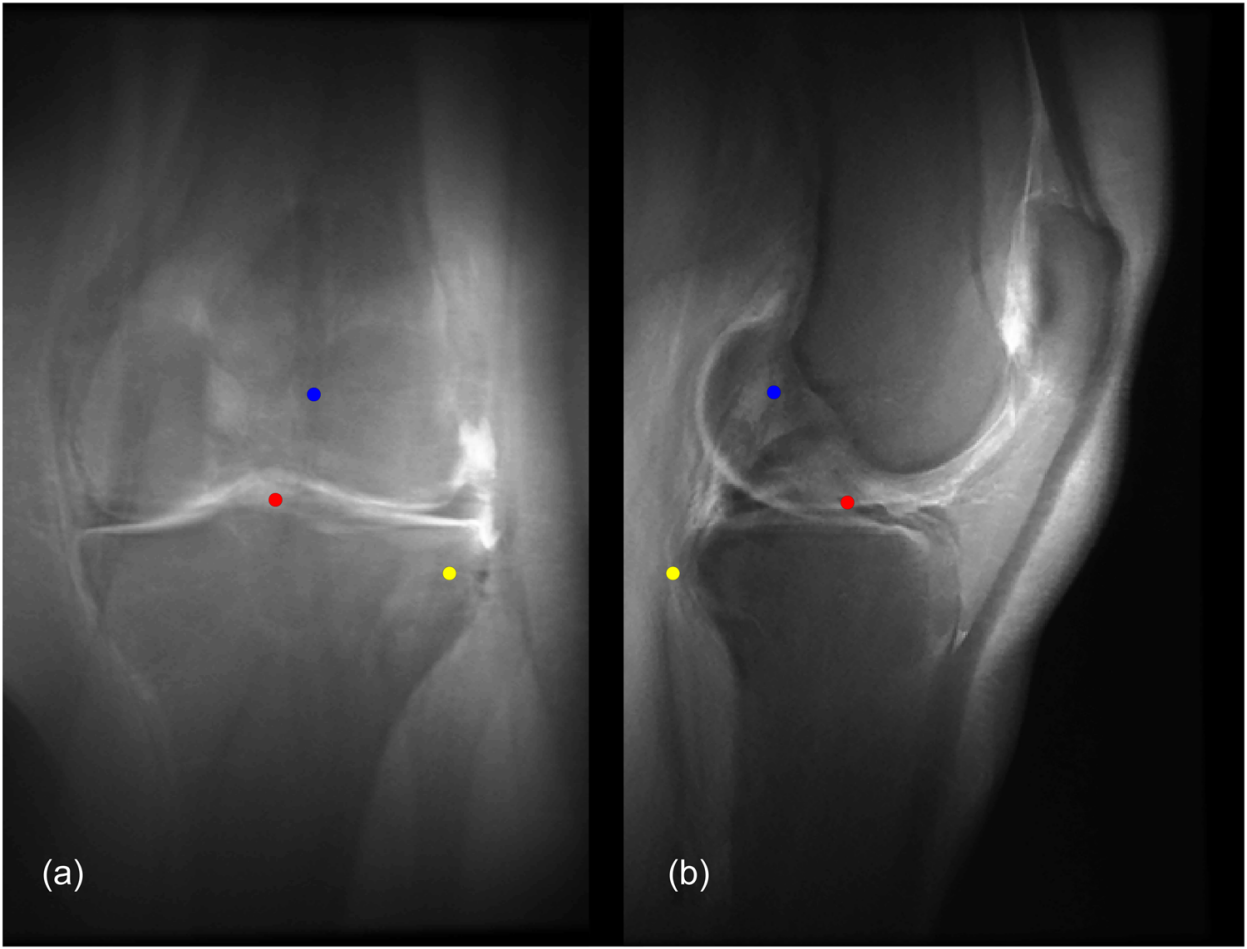
(a) Anteroposterior ‘CLASS’ view (blue point: centroid ACL femoral FP, red point: centroid ACL tibial FP, yellow point: the styloid process of fibular head); (b) Lateral ‘CLASS’ view (blue point: centroid ACL femoral FP, red point: centroid ACL tibial FP, yellow point: the styloid process of fibular head). ACL, anterior cruciate ligament; CLASS, Compressed Lateral and anteroposterior Anatomic Systematic Sequence; FP, footprint.

The adopted sequences encompassed sagittal proton density fat‐saturated isotropic 3D configurations with an isovoxel resolution of 0.6 × 0.6 × 0.6 mm^3^. FP identification was meticulously executed using the *multiplanar reformation* tool integrated within the Mimics (Materialise Mimics 17.0) software by both a board‐certified orthopaedic surgeon specialized in knee surgery and a board‐certified radiologist with proficiency in advanced musculoskeletal imaging. FP on the femoral region were delineated as shallow, deep, high and low, while those on the tibial region were characterized as anterior, posterior, medial and lateral [3]. The ACL FP was directly visualized on both the femoral and tibial sides. All demarcated points were selected at their maximal extents. Consistent with methodologies employed in the prior proof of concept study [46], a CLASS was constructed by projecting the volumetric MRI imagery alongside the computed points onto true lateral and anteroposterior views (Figure 1).

### Radiographic evaluation

**Figure 2:**
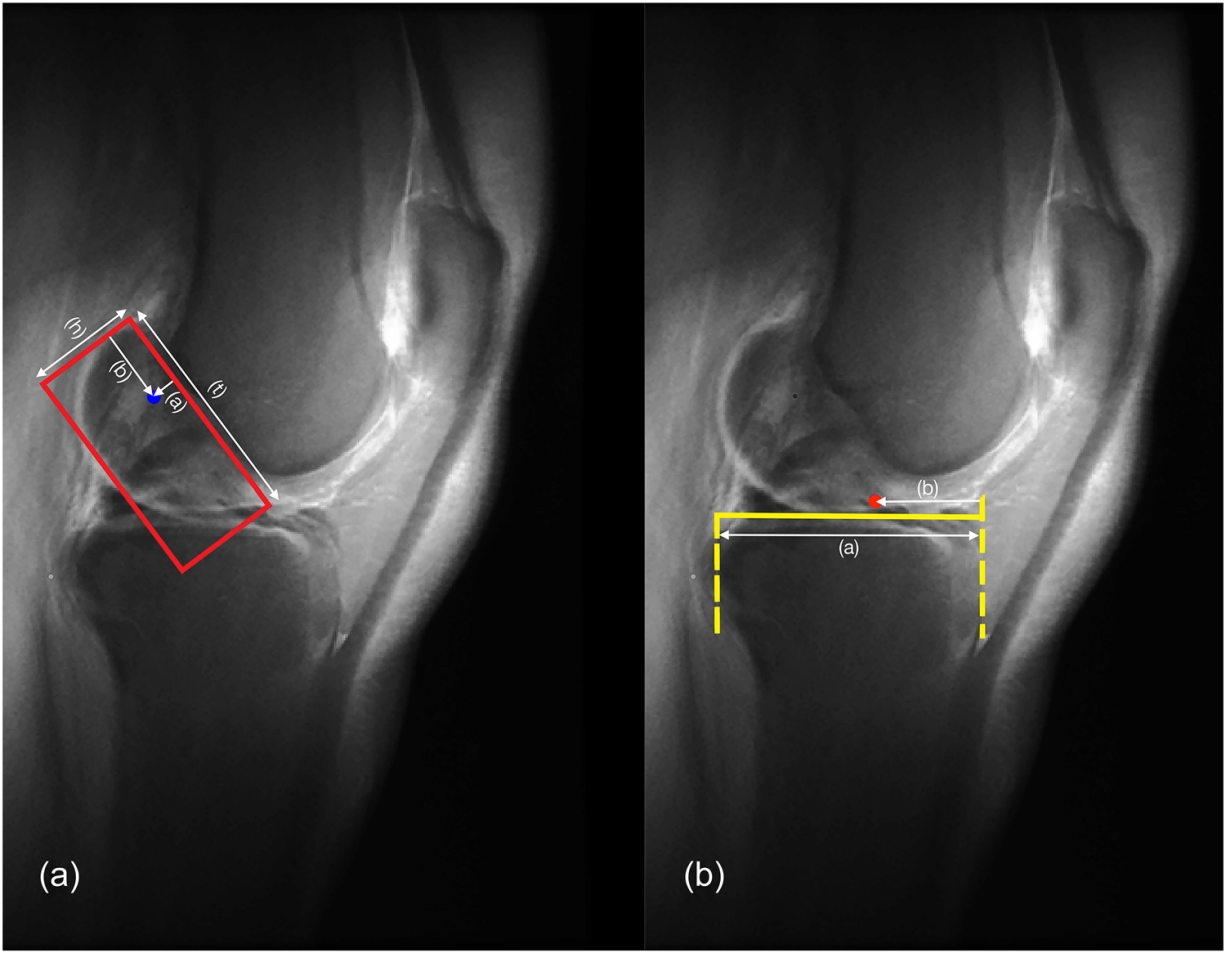
(a) Lateral ‘CLASS’ view (blue point: centroid ACL femoral FP, red square: (t) deep‐shallow distance 100%, (b) posterior edge of femoral condyles to ACL's centroid point, (h) high‐low distance 100%, (a) Blumensaat line to ACL's centroid point); (b) Lateral ‘CLASS’ view (red point: centroid ACL tibial FP, yellow line: (a) distance between tibial anterior border to posterior corner of the shelf, (b) distance between tibial anterior border and ACL's centroid point). ACL, anterior cruciate ligament; CLASS, Compressed Lateral and anteroposterior Anatomic Systematic Sequence; FP, footprint.

Using the software ImageJ, the following measurements were performed on the CLASS 2D reconstructions [40]. To identify the ACL‐FP, the coordinates were collected in the *x*‐, *y*‐ and *z*‐axes to reduce any measurement bias (Figure 2).

Measurements:

Location of femoral ACL FP:

‐ ‘sagittal femoral FP high to low ratio’ and ‘sagittal femoral FP deep to shallow ratio’: on lateral (LAT) view, the sagittal location of the ACL femoral FP deep to shallow (DS) and high to low (HL) were determined by using the reference lines according to Bernard et al. [6].
  Location of tibial ACL FP:
‐ ‘coronal tibial FP ratio’: on anteroposterior (AP) view, the distance between the lateral border of the tibial plateau to the tibial ACL FP was divided by the distance from the lateral to the medial border of the tibial plateau.
‐ ‘sagittal tibial FPratio’: on LAT view, the sagittal location of the ACL tibial FP was determined by applying the reference line according to Amis and Jakob [3].

Additional measurements on the CLASS 2D reconstructions included:

The femoral intercondylar notch roof angle (*α*) on the LAT view was determined using the longitudinal axis of the femur and the Blumensaat line. Two circles were drawn, one tangent to the Blumensaat line and the anterior and posterior femur edges and the other tangent to the proximal border of the distal circle, anterior and posterior to the femur edges. The longitudinal femoral axis was then assessed by connecting the centres of both circles. The angle between the tibial articular surface and the ACL FPs was calculated from both the AP and LAT views (‘coronal articular surface and ACL‐angle’ and ‘sagittal articular surface and ACL‐angle’). On the LAT view, the angle between the tibial articular surface and the Blumensaat's line was measured (‘sagittal articular surface’ and ‘Blumensaat's line‐angle’).

The tibial slope (*β*) was measured on the LAT view of CLASS using the longitudinal tibial axis according to Lipps et al. [26] and the articular surface. In addition, the tibial slope was calculated on the LAT view plain radiographs according to the method described by Dejour and Bonnin [11].

### Reliability and reproducibility assessment

To evaluate the intraobserver reliability and interobserver reproducibility of ACL femoral and tibial FP identification in generating CLASS images, 20 randomly selected patients were independently reassessed by the same observers. The evaluations using *x*‐, *y*‐ and *z*‐coordinates were conducted independently by both observers during two distinct time periods, separated by a 3‐week interval, to ensure consistency. Identifying details and names of the patients were anonymized to prevent recall bias. In addition, the reliability and reproducibility of the measurements were assessed using twenty randomly selected CLASS images, which were independently reassessed by the same observers. These evaluations also took place during two distinct time periods, separated by a 3‐week interval, with patient details anonymized to mitigate recall bias.

### Statistical analysis

To assess the intraobserver reliability and interobserver reproducibility, intraclass correlation coefficient (ICC), including 95% confidence intervals (CIs), was calculated based on a mean‐rating (*k* = 2), absolute agreement, two‐way mixed‐effects model. The ICC was graded as ICC < 0.20 for *slight*; 0.21–0.40 for *fair*; 0.41–0.60 for *moderate*; 0.61–0.80 for *substantial* and >0.80 for *almost perfect* agreement [32].

The Shapiro–Wilk test was utilized to assess data normality. For normally distributed data, a one‐way repeated measures analysis of variance (ANOVA), incorporating Bonferroni correction, was performed. Conversely, for non‐normally distributed data, the Friedman test was employed. A significance threshold was set at *α* = 0.05, and a test result was deemed significant when *p* < 0.05.

The observed femoral and tibial FPs were benchmarked against established references. For the femoral FPs, the references were as follows:

- DS at 30.6% and HL at 32.3% according to Iriuchishima et al. [19].
- DS at 28.6% and HL at 34.5% according to Parkar et al. [33].
- DS at 28.5% and HL at 35.2% according to Piefer et al. [36].

For the tibial FPs, the references were as follows:

- coronal lateral to medial at 53% according to Pinczewski et al. [38].
- sagittal anterior to posterior at 43% according to Amis and Jakob [3].
- sagittal anterior to posterior at 40.9% according to Cho et al. [7].

The statistical analysis was carried out using IBM SPSS Version 26.0 (SPSS Inc). The study was carried out following the World Medical Association Declaration of Helsinki. According to federal law, our project was approved by the local ethical committee (CER‐VD 2021‐00818).

## RESULTS

A total of 82 knee MRI scans (49 females, 33 males; mean age 34 years, range 18–65) were analyzed. The descriptive statistics for the measurements are summarized in Table 1.

**Table 1:** Descriptive statistics (mean [SD; range]).

	*N* = 82
Age (years)	34 (n.a.; 18–65)
ACL FP	
Sagittal femoral FP DS	
Absolute (mm)	15.6 (±2.2; 11.1–20.5)
Ratio (%)	33.5 (±3.4; 24–43)
Sagittal femoral FP HL	
Absolute (mm)	5.5 (±1.2; 3.2–8.7)
Ratio (%)	23.4 (±4.7; 14–36)
Coronal tibial FP (lateral to medial)	
Absolute (mm)	40.7 (±3.7; 32.1–49.5)
Ratio (%)	52.8 (±2.4; 47–57)
Sagittal tibial FP (anterior‐posterior)	
Absolute (mm)	22.0 (±2.7; 15.4–31.2)
Ratio (%)	42.3 (±3.2; 35–51)
Additional measurements	
α‐angle (°)	38.0 (±4.9; 27–48)
β‐CLASS (°)	86.7 (±3.3; 79–94)
β‐X‐Ray (°)	82 (±3.1; 76–89)
Coronal articular surface and ACL angle (lateral to medial; °)	68.3 (±5.3; 56–80)
Sagittal articular surface and ACL angle (°)	55.2 (±4.1; 45–64)
Sagittal articular surface and Blumensaat's line angle (°)	53.4 (±5.3; 44–66)

Abbreviations: ACL, anterior cruciate ligament; CLASS, Compressed Lateral and anteroposterior Anatomic Systematic Sequence; DS, deep to shallow; FP, footprint; HL, high to low; SD, standard deviation.

### Reliability and reproducibility

Generating the CLASS images with the centroid ACL FPs showed an ‘almost perfect’ reliability and reproducibility of localizing the same centroid ACL FP (Table 2). The reliability and reproducibility of the different measurements showed an ‘almost perfect’ reliability and reproducibility, except for the tibial slope angle (β‐CLASS‐angle) which showed a ‘substantial’ reliability for one observer (Table 3).

**Table 2:** Intra‐rater reliability and inter‐rater reproducibility generating CLASS (mean ICC; 95% CI).

CLASS	Reliability Reader 1	Reliability Reader 2	Reproducibility Reader 1 versus Reader 2
Coronal femoral FP			
*x*‐axis	0.999 (0.997–1.000)	0.993 (0.982–0.997)	0.996 (0.992–0.998)
y‐axis	1.000 (1.000–1.000)	0.970 (0.678–0.992)	0.985 (0.948–0.995)
Coronal tibial FP			
*x*‐axis	0.998 (0.994–0.999)	0.998 (0.994–0.999)	0.998 (0.997–0.999)
*y*‐axis	1.000 (1.000–1.000)	1.000 (1.000–1.000)	1.000 (1.000–1.000)
Sagittal femoral FP			
*y*‐axis	1.000 (1.000–1.000)	0.970 (0.678–0.992)	0.985 (0.948–0.995)
*z*‐axis	0.998 (0.994– 0.999)	0.978 (0.862–0.993)	0.990 (0.971–0.996)
Sagittal tibial FP			
*y*‐axis	1.000 (1.000–1.000)	1.000 (1.000–1.000)	1.000 (1.000–1.000)
*z*‐axis	0.997 (0.978–0.999)	0.998 (0.985–0.999)	0.998 (0.995–0.999)

Abbreviations: 95% CI, 95% confidence interval; CLASS, Compressed Lateral and anteroposterior Anatomic Systematic Sequence; FP, footprint; ICC, intraclass correlation coefficient; x, coordinates medial‐lateral; y, coordinates cranial‐caudal; z, coordinates anterior‐posterior.

**Table 3:** Intra‐rater reliability and inter‐rater reproducibility of measurements using CLASS (mean ICC; 95% CI).

Measurements	Reliability Reader 1	Reliability Reader 2	Reproducibility Reader 1 versus Reader 2
Sagittal femoral FP HL ratio	0.896 (0.617–0.964)	0.952 (0.881–0.981)	0.895 (0.747–0.958)
Sagittal femoral FP DS ratio	0.968 (0.920–0.987)	0.854 (0.619–0.944)	0.890 (0.785–0.951)
Coronal tibial FP ratio (lateral to medial)	0.964 (0.908–0.986)	0.939 (0.846–0.976)	0.865 (0.735–0.941)
Sagittal tibial FP ratio	0.852 (0.634–0.941)	0.883 (0.707–0.954)	0.862 (0.727–0.939)
Additional measurements			
α‐angle	0.994 (0.890–0.998)	0.984 (0.953–0.994)	0.838 (0.683–0.928)
β‐CLASS	0.955 (0.887–0.982)	0.972 (0.930–0.989)	0.791 (0.590–0.908)
β‐X‐ray	0.976 (0.939–0.990)	n/a	n/a
Coronal articular surface and ACL angle (lateral to medial)	0.995 (0.979–0.998)	0.992 (0.980–0.997)	0.956 (0.889–0.982)
Sagittal articular surface and ACL angle	0.982 (0.940–0.993)	0.982 (0.955–0.993)	0.915 (0.833–0.962)
Sagittal articular surface and Blumensaat's line angle	0.997 (0.993–0.999)	0.978 (0.942–0.991)	0.894 (0.792–0.953)

Abbreviations: 95% CI, 95% confidence interval; ACL, anterior cruciate ligament; CLASS, Compressed Lateral and anteroposterior Anatomic Systematic Sequence; DS, deep to shallow; FP, footprint; HL, high to low; ICC, intraclass correlation coefficient; DS, deep to shallow; FP, footprint.

**Graph 1:**
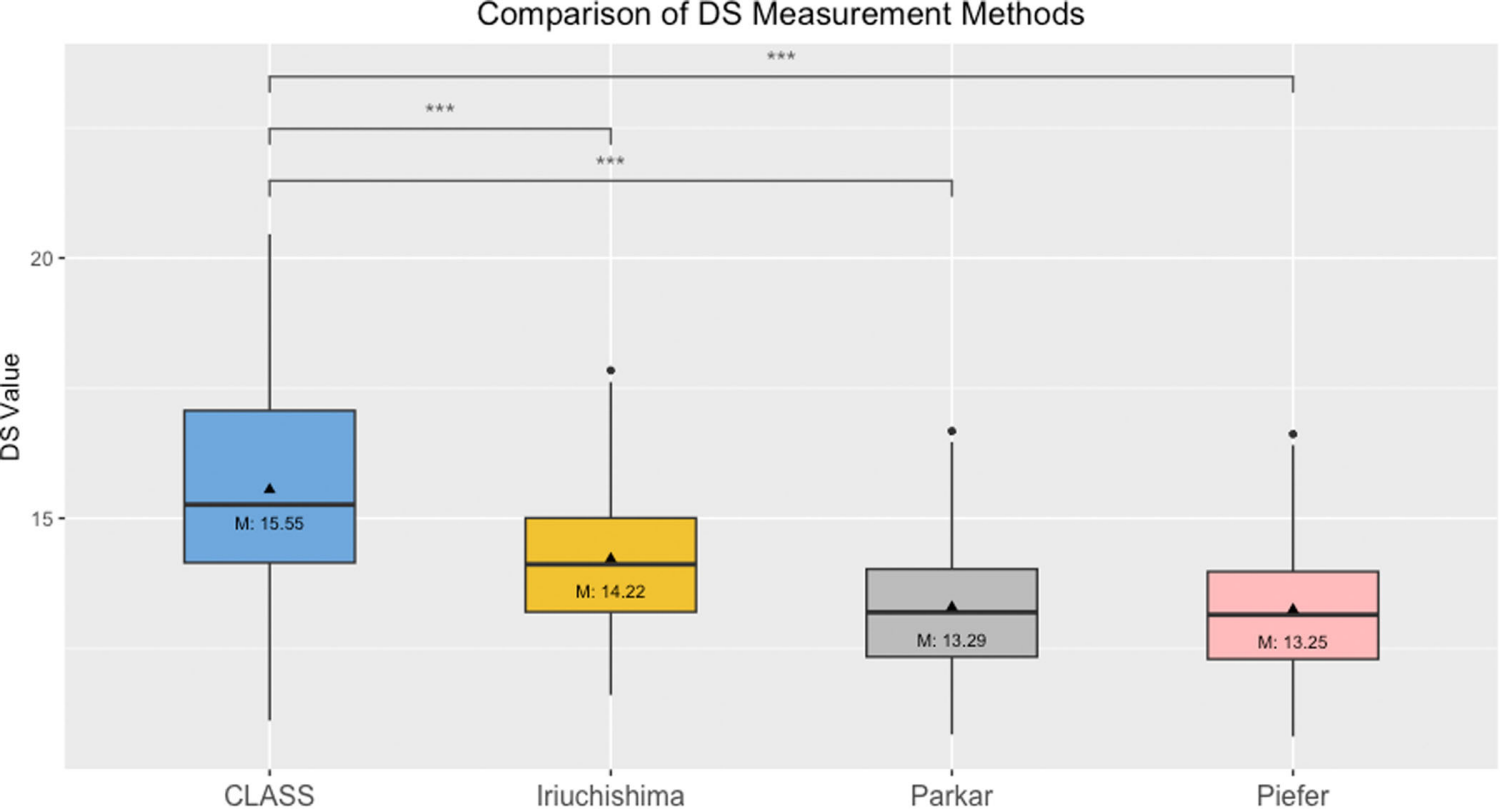
One‐way ANOVA multiple measurements for deep‐shallow position comparing the values of ‘CLASS’ to the values of ‘Iriuchishima’, ‘Parkar’ and ‘Piefer’. ANOVA, analysis of variance; CLASS, Compressed Lateral and anteroposterior Anatomic Systematic Sequence; DS value, deep to shallow value in mm; M, mean in mm; (***) *p* < 0.000 = significant.

**Graph 2:**
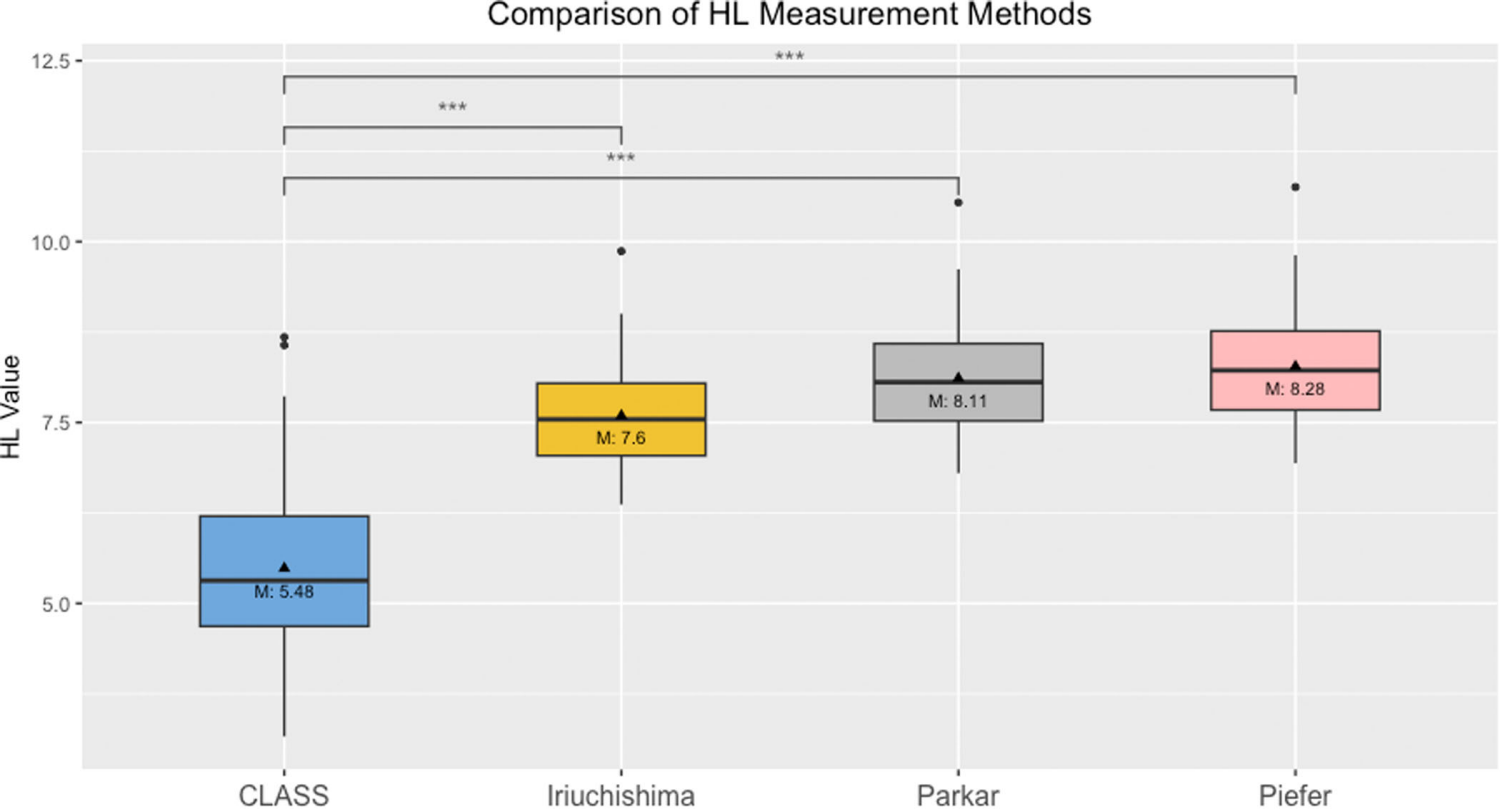
One‐way ANOVA multiple measurements for high‐low position comparing the values of ‘CLASS’ to the values of ‘Iriuchishima’, ‘Parkar’ and ‘Piefer’. ANOVA, analysis of variance; CLASS, Compressed Lateral and anteroposterior Anatomic Systematic Sequence; HL value, high to low value in mm; M, mean in mm; (***) *p* < 0.000 = significant).

**Figure 3:**
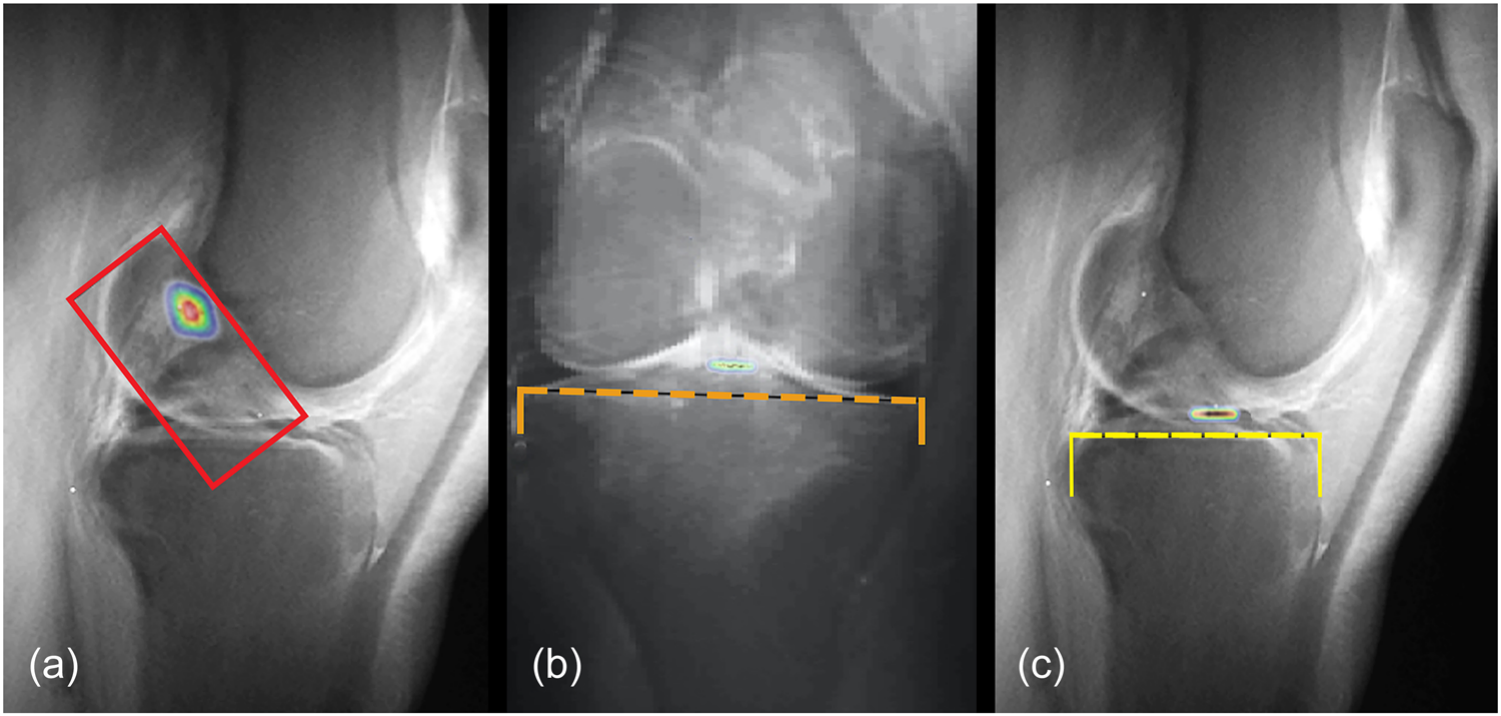
(a–c) Heatmap of the anterior cruciate ligament FPs; (a) sagittal CLASS with femoral heatmap of ACL FP, red square: deep to shallow and high to low according to Bernard et al. [6]; (b) coronal CLASS with tibial heatmap of ACL FP, orange dashed line: distance between the outer limits of the lateral and medial tibial plateau according to Pinczewski et al. [38]; (c) sagittal CLASS with tibial heatmap of ACL FP, yellow dashed line: distance between tibial anterior border to posterior corner of the shelf according to Amis and Jakob [3]. ACL, anterior cruciate ligament; CLASS, Compressed Lateral and anteroposterior Anatomic Systematic Sequence; FP, footprint.

The Shapiro–Wilk test confirmed that all measurements conformed to a normal distribution. The one‐way ANOVA test results indicated a significant variation among the groups of DS (*F* = 3879.472, *p* < 0.000, *η*² = 0.990) and HL (*F* = 5043.230, *p* < 0.000, *η*² = 0.992). Subsequent post hoc analyses using the Bonferroni correction highlighted significant disparities in the DS and HL positions between Group ‘CLASS’ and the groups of ‘Iriuchishima’, ‘Parkar’ and ‘Piefer’ (Table 4). These findings are presented in Graphs 1, 3 and 2. To enhance visual interpretation, maps of the resultant tibial and femoral ACL FPs were created, as depicted in Figure 1, 3.

**Table 4:** One‐way ANOVA comparing femoral ACL FPs (mean; standard deviation).

Groups		*p*
Deep‐shallow		
Iriuchishima	CLASS	
14.2 ± 1.4	15.6 ± 2.2	<0.000*
Parkar	CLASS	
13.3 ± 1.3	15.6 ± 2.2	<0.000*
Piefer	CLASS	
13.3 ± 1.3	15.6 ± 2.2	<0.000*
High‐low		
Iriuchishima	CLASS	
7.6 ± 0.7	5.5 ± 1.2	<0.000*
Parkar	CLASS	
8.1 ± 0.7	5.5 ± 1.2	<0.000*
Piefer	CLASS	
8.3 ± 0.7	5.5 ± 1.2	<0.000*

*Note*: Mean in mm.

Abbreviations: ACL, anterior cruciate ligament; ANOVA, analysis of variance; CLASS, Compressed Lateral and anteroposterior Anatomic Systematic Sequence; DS, deep to shallow; FP, footprint; HL, high to low.

* *p* < 0.000 = significant.

The one‐way ANOVA test revealed no significant differences when comparing the tibial FPs of CLASS towards the groups of Pinczewski et al. [38], Amis and Jakob [3] and Cho et al. [7].

A post hoc power analysis was conducted using G*Power software. With a sample size of 82 and an effect size *f* of 1.27, the achieved power was 1.00 (critical *F* = 2.63, df = 364 and noncentrality parameter *λ* = 591.89).

## DISCUSSION

The most important finding of this study was the validation of the CLASS MRI technique for identifying ACL FPs, demonstrating its high reliability and reproducibility in providing accurate patient‐specific data. The CLASS method compresses 3D‐MRI data into lateral and anteroposterior views, allowing for accurate projection of femoral and tibial ACL FP centroids. The study's strength lies not only in its large sample size and high‐reliability metrics but also in its potential for a patient‐specific approach, addressing the individual anatomical variations often overlooked in standardized techniques. By benchmarking this study's results against established methods, both the consistency and the unique insights offered by CLASS, providing a new, comprehensive dataset of ACL insertion site characteristics, are highlighted. This novel approach represents a significant step forward in bridging the gap between advanced imaging techniques and the anatomical details necessary for precise ACL reconstruction.

Over the past 30 years, numerous authors have focused their efforts on identifying the anatomical landmarks of the ACL FP to improve the surgical precision, reproducibility and outcomes of ACL reconstructions. However, despite numerous systematic reviews, a unanimous consensus on the optimal graft position remains difficult to achieve [21, 33, 36, 48].

The quadrant method was introduced as a simple and reproducible tool to locate and quantify the femoral ACL FP [6]. Notable contributions by Amis and Zavras in 1995 (DS 38% and HL 20%), and Bernard et al. in 1996 (DS 24.8% and HL 28.5%), presented their respective findings related to ACL FP [4, 6]. It should be noted that the HL position described by Amis and Zavras is a percentage that relates to the total length of the Blumensaat line and not to the length of the Blumensaat line to the femoral condyle. However, recent systematic reviews have revealed discrepancies in these metrics [19, 33, 36, 48]. These results presented in this study are in contradiction with the already published data. One possible explanation lies in small differences in tracing the Blumensaat line. Iriuchishima et al. [20] identified three distinct types of shape of the Blumensaat line, emphasizing that the choice of line can potentially alter ACL FP identification. Consequently, for a small or large hill type of the Blumensaat, the two most distal points are recommended to be chosen when drawing the line. Yahagi et al. [49] explained the differences in the application of the quadrant method, which supports the different results observed in this study. The CLASS technique, given this anatomical variability, could potentially optimize femoral positioning, especially in the HL context. However, intraoperative validation remains imperative.

Previous meta‐analyses [33, 36, 48] correlated anatomic identification with either conventional radiography or 3D computed tomography. Such modalities can lead to a bias in data collection, as it is possible that small deviations may occur despite the same measurement method [10, 12, 24, 30, 35]. Some of these studies performed their analysis using only the lateral condyle with a marked ACL FP [9, 10, 24, 45, 47]. It is not known if the true‐lateral view is altered when only the lateral femoral condyle is used compared to the entire distal femur. Unlike this, the CLASS approach uses unaltered MRI data, compressing the data into AP and LAT views, thereby minimizing biases.

The CLASS MRI technique revealed significant variations in ACL FP locations compared to previous studies, particularly in the DS and HL positions. This finding is supported by recent morphological studies that measured a cross‐sectional area of the femoral FP ranging from 60 to 130 mm² [31], with similar high variability observed for the tibial FP. These discrepancies raise questions about the accuracy of anatomical reference points or FP locations reported in earlier research. Concerning the tibial ACL FP in the sagittal plane, the results of this study are consistent with the systematic analysis [33] and the results presented by Amis and Jakob [3]. In this regard, however, it should be noted, as mentioned above, that Amis and Jakob [3] did not use exactly the same manner as Bernard et al. [6] to calculate the HL position. The results regarding the location of the ACL FP in the latero‐medial coronal plane are consistent with the existing literature [12, 24, 37, 45, 47]. Further potential causes include methodological differences, with CLASS using high‐resolution 3D MRI (0.6 × 0.6 × 0.6 mm^3^ isovoxel resolution) compressed into 2D views, potentially offering more precise FP identification. The study's large sample size may have better captured inherent anatomical variability, while differences in reference point interpretation, especially of the Blumensaat line, could contribute to the observed variations [20]. The predominantly young, uninjured cohort used in this study differs from previous research populations, possibly accounting for some discrepancies. These findings underscore the importance of patient‐specific approaches in ACL reconstruction and suggest that standardized techniques based on previous studies may not always achieve optimal graft positioning. The implications for tunnel placement, graft isometry and overall surgical outcomes are significant, potentially necessitating a reevaluation of current ACL reconstruction techniques [31].

Improper placement of femoral, tibial, or both tunnels can lead to suboptimal results, discomfort, reduced mobility or an increased risk of ACL graft rupture [14, 17, 38, 42, 44]. CLASS offers significant clinical relevance for accurate anatomical ACL reconstruction by providing surgeons with patient‐specific ACL FP locations, allowing for personalized preoperative planning and more precise intraoperative tunnel placement. By transferring the femoral and tibial ACL‐FP to AP and LAT views using the CLASS algorithm, surgeons can use these projections as benchmarks for tunnel placement under fluoroscopic guidance [23, 28, 39, 41], potentially improving accuracy. The method's ability to address individual anatomical variability makes it particularly valuable for both primary and revision ACL surgeries, where standardized techniques may fall short. Furthermore, CLASS has the potential to integrate with advanced technologies such as navigation systems and augmented reality interfaces, enhancing surgical precision. As an educational tool, it can aid in training young surgeons to identify correct tunnel positioning. These future developments need to be explored and tested in further studies, but overall, CLASS MRI represents a promising advancement in orthopaedic surgery, offering a reliable and reproducible method for improving the accuracy and outcomes of ACL reconstructions.

There are some limitations to this study. Identification has only been performed using MRIs of a healthy population and only five patients older than 50 years were included. The authors did not consider this as a negative issue as the majority of patients requiring an ACL reconstruction procedure are below 40 years of age. Furthermore, CLASS has not yet been validated using cadaver studies. Nevertheless, this study demonstrated that ACL FP localization could be reliably and reproducibly performed by an experienced musculoskeletal radiologist and an experienced orthopaedic knee surgeon. In addition, a cadaveric knee may have altered or aged anatomy that could have led to different results [29]. Future studies should prioritize determining the exact correlation between the CLASS algorithm, the anatomical structures and the fluoroscopic images.

## CONCLUSION

The CLASS MRI sequence is a reliable and reproducible method for identifying ACL FPs and offers significant potential for improving the precision of ACL reconstructions. The observed variability in the location of the ACL FP underlines the importance of a patient‐specific surgical approach. Thus, careful preoperative planning proves essential to enable customized ACL reconstructions based on patient‐specific anatomical variations.

## AUTHOR CONTRIBUTIONS

**Grégoire Thürig**: Methodology; formal analysis; investigation; writing—original draft preparation; writing—review and editing. **Raúl Panadero‐Morales**: Methodology; formal analysis; investigation; resources. **Daniel Petek**: Methodology; formal analysis; investigation; writing—review and editing; supervision. **Joseph Schwab**: Methodology; formal analysis; investigation; writing—review and editing. **Marc Berrera Usó**: Formal analysis; investigation; writing—review and editing. **Julien Galley**: Formal analysis; investigation. **Alexander Heimann**: Formal analysis; investigation. **Moritz Tannast**: Writing—review and editing; resources; supervision.

## CONFLICT OF INTEREST STATEMENT

The authors declare no conflicts of interest.

## ETHICS STATEMENT

According to federal law, our project was approved by the local ethical committee (CER‐VD 2021‐00818). This study utilized anonymized patient data from MRI scans and radiographs that were already performed for clinical purposes. As per the ethical guidelines and federal law, patient consent was not required due to the retrospective nature of the study and the use of fully anonymized data.

## Data Availability

The data that support the findings of this study are available on request from the corresponding author. The data are not publicly available due to privacy or ethical restrictions.
